# Phase II study of second-line therapy with DTIC, BCNU, cisplatin and tamoxifen (Dartmouth regimen) chemotherapy in patients with malignant melanoma previously treated with dacarbazine

**DOI:** 10.1054/bjoc.2000.1141

**Published:** 2000-06

**Authors:** D J Propper, J P Braybrooke, N C Levitt, K O'Byrne, K Christodoulos, C Han, D C Talbot, T S Ganesan, A L Harris

**Affiliations:** ICRF Medical Oncology Unit, Churchill Hospital, Headington, Oxford, OX3 7LJ, UK

**Keywords:** melanoma, DTIC, Dartmouth regimen

## Abstract

This study assessed response rates to combination dacarbazine (DTIC), BCNU (carmustine), cisplatin and tamoxifen (DBPT) chemotherapy in patients with progressive metastatic melanoma previously treated with DTIC, as an evaluation of DBPT as a second-line regimen, and as an indirect comparison of DBPT with DTIC. Thirty-five consecutive patients received DBPT. The patients were divided into two groups. Group 1 comprised 17 patients with progressive disease (PD) on DTIC + tamoxifen therapy who were switched directly to DBPT. Group 2 comprised 18 patients not immediately switched to DBPT and included patients who had either a partial response (PR; one patient) or developed stable disease (SD; four patients) with DTIC, or received adjuvant DTIC (nine patients). All except four patients had received tamoxifen at the time of initial DTIC treatment. Median times since stopping DTIC were 22 days (range 20–41) and 285 days (range 50–1240) in Groups 1 and 2 respectively. In Group 1, one patient developed SD for 5 months and the remainder had PD. In Group 2, there were two PRs, four patients with SD (4, 5, 6, and 6 months), and 11 with PD. These results indicate that the DBPT regimen is not of value in melanoma primarily refractory to DTIC. There were responses in patients not directly switched from DTIC to DBPT, suggesting combination therapy may be of value in a small subgroup of melanoma patients. © 2000 Cancer Research Campaign


British Journal of Cancer (2000) **82**(11), 1759–1763
© 2000 Cancer Research Campaign
DOI: 10.1054/ bjoc.2000.1141, available online at http://www.idealibrary.com on IDEAL®

### Phase II study of second-line therapy with DTIC, BCNU, cisplatin and tamoxifen (Dartmouth regimen) chemotherapy in patients with malignant melanoma previously treated with dacarbazine

**DJ Propper, JP Braybrooke, NC Levitt, K O'Byrne, K Christodoulos, C Han, DC Talbot, TS Ganesan and AL Harris**

ICRF Medical Oncology Unit, Churchill Hospital, Headington, Oxford OX3 7LJ, UK

**Summary** This study assessed response rates to combination dacarbazine (DTIC), BCNU (carmustine), cisplatin and tamoxifen (DBPT) chemotherapy in patients with progressive metastatic melanoma previously treated with DTIC, as an evaluation of DBPT as a second-line regimen, and as an indirect comparison of DBPT with DTIC. Thirty-five consecutive patients received DBPT. The patients were divided into two groups. Group 1 comprised 17 patients with progressive disease (PD) on DTIC + tamoxifen therapy who were switched directly to DBPT. Group 2 comprised 18 patients not immediately switched to DBPT and included patients who had either a partial response (PR; one patient) or developed stable disease (SD; four patients) with DTIC, or received adjuvant DTIC (nine patients). All except four patients had received tamoxifen at the time of initial DTIC treatment. Median times since stopping DTIC were 22 days (range 20–41) and 285 days (range 50–1240) in Groups 1 and 2 respectively. In Group 1, one patient developed SD for 5 months and the remainder had PD. In Group 2, there were two PRs, four patients with SD (4, 5, 6, and 6 months), and 11 with PD. These results indicate that the DBPT regimen is not of value in melanoma primarily refractory to DTIC. There were responses in patients not directly switched from DTIC to DBPT, suggesting combination therapy may be of value in a small subgroup of melanoma patients. © 2000 Cancer Research Campaign

**Keywords:** melanoma; DTIC; Dartmouth regimen

In patients with metastatic melanoma, the most effective chemotherapeutic single agent is DTIC. Response rates to this drug, often given in combination with tamoxifen, are around 20% (Hill et al, 1984; Mastrangelo et al, 1992). After DTIC chemotherapy, almost all patients eventually develop progressive disease. The place of second-line chemotherapy in such patients is unclear. Low response rates have been reported in small numbers of DTIC-resistant patients, with combination carboplatin and cytosine arabinoside (Bajetta et al, 1995), and combination vinblastine, bleomycin and methotrexate (Porcile et al, 1979). In addition, there are trials of various chemotherapies which included small numbers of patients who had already received DTIC, and some of these retreated patients, although the proportion is not clear, responded (Quagliana et al, 1984; Johnson et al, 1985; Mulder et al, 1990; Rusciani et al, 1997).

Melanoma response rates of above 40% have been reported for combination chemotherapy regimens (McClay and McClay, 1996). One of the most widely used is DTIC, BCNU, cisplatin and tamoxifen (DBPT) – the Dartmouth regimen. Because of reported high response rates, it has been suggested that this regimen should be used in preference to the more easily administered and less toxic DTIC regimen (Reintgen and Saba, 1993; McClay and McClay, 1994). Yet there is uncertainty as to whether this regimen is superior to DTIC, because of reported response rates similar to DTIC (Johnston et al, 1998; Margolin et al, 1998). Furthermore, recent randomized comparison of the two regimens does not indicate significantly greater activity for DBPT (Saxman et al, 1999).

If the DBPT regimen is superior to DTIC, then we reasoned that it might prove effective in a proportion of patients who were refractory to DTIC therapy, and it might also benefit patients who had received DTIC treatment previously, and with subsequent disease progression required further therapy. This approach would also provide an indirect comparison of the two regimens. In this trial, we therefore assessed the effect of the DBPT regimen on patients with metastatic melanoma who had previously received DTIC. We assessed the effect in two groups of patients: those with progressive disease refractory to DTIC who were switched directly to the DBPT regimen, and those who were not immediately switched to the DBPT regimen. This latter group included patients who had either previously responded or attained stable disease with DTIC treatment, or patients who had received DTIC as adjuvant therapy.

Received 22 March 1999
Revised 11 October 1999
Accepted 17 February 2000

### PATIENTS AND METHODS

### Eligibility criteria

Thirty-five consecutive patients with histologically or cytologically proven metastatic malignant melanoma with objective evidence of progressive disease were enrolled in the trial. Other eligibility criteria included: Eastern Co-operative Oncology Group (ECOG) performance status of 0–2, white cell count greater than $3.0 \times 10^9$ L$^{-1}$, platelet count greater than $100 \times 10^9$ L$^{-1}$, normal

**Table 1** Details of patients at start of DBPT treatment

| | Direct switch DTIC to DBPT | Delayed switch DTIC to DBPT | Statistical differences between groups (*P*-value) |
|---|---|---|---|
| No. treated | 17 | 18 | |
| M/F | 5/12 | 12/6 | < 0.03 |
| Age, years | 46 (29–81) | 54 (35–72) | NS |
| Performance status | 0 (0–3) | 0 (0–3) | NS |
| Time since stopping DTIC | 22 days (20–41) | 285 days (50–1240) | < 0.0001 |
| Previous response to DTIC | | | NS |
| PD | 17 | 4 | |
| SD | | 4 | |
| PR | | 1 | |
| Adjuvant | | 9 | |
| No. of previous cycles of DTIC | | | < 0.001 |
| 1 | 2 | 1 | |
| 2 | 7 | 2 | |
| 3 | 8 | 2 | |
| 4 | | 12 | |
| 5 | | 1 | |
| Site of disease | | | NS |
| Lymph node | 4 | 8 | |
| Skin/muscle | 11 | 6 | |
| Lung | 7 | 12 | |
| Liver | 3 | 4 | |
| Intra-abdominal | 2 | 3 | |
| Bone | 2 | 4 | |
| Stage of disease: all stage IV: | | | NS |
| Confined to skin/lymph nodes | 4 | 2 | |
| Beyond skin/lymph nodes | 13 | 16 | |

Median and range values shown. PD: progressive disease; SD: stable disease; PR: partial response. NS: not significant renal and hepatic function, the absence of brain metastases, the presence of measurable or evaluable disease, and informed consent. All patients had previously received DTIC chemotherapy. All but four of the patients had received concomitant tamoxifen (20 mg day$^{-1}$) commencing with the first cycle of DTIC and continuing for 21 days following the last DTIC treatment. Of the remaining four patients, three had received DTIC and concomitant hydroxyurea as part of a study assessing the effects of hydroxyurea on DNA excision-repair during DTIC treatment (Philip et al, 1994). The remaining patient had received DTIC alone. No patients had received other cytotoxic chemotherapy. Four patients had received the protein kinase C partial antagonist bryostatin as part of a trial (Propper et al, 1998) and two patients had received low dose weekly interferon γ (100 μg m$^{-2}$) as part of an ongoing trial.

### Assessment of disease response and toxicity

Evaluable and measurable disease sites were assessed before entering the study by physical examination, plain radiography and computerized tomography and/or magnetic resonance imaging where appropriate. Patients were reviewed by a physician before each cycle of chemotherapy, and new signs, symptoms and performance status (ECOG) documented. Before each cycle of chemotherapy a full blood count with a differential white cell count, serum biochemistry and chest X-ray were performed. Further imaging investigations for the purposes of tumour measurement were repeated after every two cycles of treatment, or at the time of suspected disease progression. National Cancer Institute Common Toxicity Criteria (NCI-CTC) were used to grade any toxicities.

Standard WHO criteria for objective response assessment were employed. Partial response was defined as a 50% or greater reduction in the sum of the products of the largest perpendicular diameters of all measurable disease sites. Progressive disease was indicated by a greater than 25% increase in the size of at least one measurable lesion, or the appearance of a new lesion. Stable disease was defined as an increase in disease measurements of less than 25% or a decrease by less than 50%. Patients with progressive disease were withdrawn from the study.

### Drug regimen

The drug regimen was as previously described (Del Prete et al, 1984). Briefly this comprised cisplatin 25 mg m$^{-2}$ day$^{-1}$ and DTIC 220 mg m$^{-2}$ day$^{-1}$, both on days 1–3, given in three weekly cycles and BCNU 100 mg m$^{-2}$ on day 1 of cycles 1, 3 and 6. Tamoxifen 160 mg was given as a loading dose immediately before the first cycle of chemotherapy and continued at 20 mg day$^{-1}$ until 3 weeks after the last.

### Statistical analysis

To ensure a low probability of erroneously rejecting a treatment that is active in 20% of patients, at least 14 patients were treated,

 © 2000 Cancer Research Campaign

**Table 2** Responses to DBPT treatment

| | Direct switch DTIC to DBPT | Delayed switch DTIC to DBPT | Statistical difference between groups (*P*-value) |
|---|---|---|---|
| No. treated | 17 | 18 | |
| Response to DBPT | | | NS |
| CR | 0 | 0 | |
| PR | 0 | 2 (4.5 and 7 months) | |
| SD | 1 (5 months) | 4 (4, 5, 6 and 6 months) | |
| PD | 16 | 12 | |
| Median no of response to (range) | 2.5 (1–4) | 4 (1–6) | NS |
| Median time to DBPT treatment failure | 44 days 95% CI 42–75 | 92 days 95% CI 39–118 | NS |
| Median survival from commencing DBPT | 109 days 95% CI 78–234 | 143 days 95% CI 127–223 | NS |

CR = complete response; PR = partial response; SD = stable disease; PD = progressive disease. CI = confidence intervals.

**Table 3** Survival data from first presentation of melanoma and from commencing DTIC treatment

| | Direct switch DTIC to DBPT | Delayed switch DTIC to DBPT | Statistical difference between groups (p value) |
|---|---|---|---|
| No. treated | 17 | 18 | |
| Survival from primary diagnosis (years) | 1.6 (0.4–2.5) | 2.8 (0.7–8.8) | NS |
| Relapse free survival from primary diagnosis (years) | 1 (0.3–21) | 1.3 (0.2–6) | NS |
| Survival from commencing DTIC all patients (years) | 0.5 (0.2–2) | 1.4 (0.6–4.3) | < 0.0001 |
| Survival from commencing non-adjuvant DTIC (years) | 0.5 (0.2–2) | 1.5 (0.9–4.2) | < 0.0004 |

Median and range values shown. Data compared for all patients and (bottom line) for patients who received DTIC for metastatic disease = 17 patients in direct switch group, and nine patients in delayed switch group.

according to previously described principles (Gehan, 1961). Survival analysis by Kaplan–Meier survival curves were compared using log-rank tests. All other data where time was a variable were compared by two-sample Wilcoxon rank-sum tests. Categorical data were compared by $\chi^2$ tests. *P*-values ≤ 0.05 were considered significant.

### RESULTS

Thirty-five patients with progressive metastatic malignant melanoma previously treated with DTIC were enrolled into the study. Their previous treatment details and characteristics are shown in Table 1. Overall there were two PRs and five SDs, suggesting the regimen is of low efficacy as a second-line treatment. In order to examine this further, the patients were analysed in two groups. The first group comprised patients who were switched directly from DTIC treatment to the DBPT regimen because of progressive disease refractory to DTIC. All these patients had received DTIC with tamoxifen. The median time since stopping DTIC was 22 days (range 20–41 days). There were no responses to the DBPT regimen (Table 2); 16 patients had PD, and one attained SD for 5 months. This latter patient had slow growing disease confined to the skin before commencing the DBPT regimen. The median number of courses of DBPT chemotherapy given was 2.5 (range 1–4) and the median survival from the time of commencing DBPT chemotherapy was 109 days (Table 2).

The second group comprised 18 patients who were not immediately changed from DTIC chemotherapy to the DBPT regimen (Table 1). Fourteen of these 18 patients had received tamoxifen at the time of initial DTIC therapy. This group included patients who had either attained a PR (one patient) or attained SD (four patients) with initial DTIC treatment, or received DTIC as adjuvant therapy (nine patients) or had PD with DTIC and then received other biological treatments (four patients). The median time since stopping DTIC was 285 days (range 50–1240). In response to DBPT, two patients, who had received DTIC (and tamoxifen) in the adjuvant setting, attained PRs (4.5 and 7 months), and four patients attained SD (4, 5, 6 and 6 months). Of these latter four patients, one had received DTIC as adjuvant therapy, one had attained SD and two had PD in response to DTIC. The remaining 12 patients had PD in response to DBPT (Table 2). Of the four patients who

*© 2000 Cancer Research Campaign* 

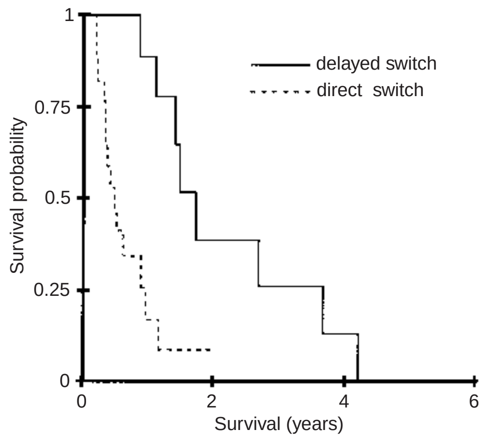

**Figure 1:** Survival probability from commencing DTIC for metastatic disease. Kaplan–Meier survival curves shown from start of DTIC therapy. Data shown only for patients who received DTIC as initial therapy for metastatic disease = 17 in direct switch group and 9 in delayed switch group. Survival compared by Logrank tests

**Table 4** NCI-CTC toxicities associated with DBPT regimen

| | No. of patients affected | |
|---|---|---|
| **Toxicity** | **Grade 3** | **Grade 4** |
| Neutropenia | 3 | 1 |
| Thrombocytopenia | 2 | 0 |
| Nausea/vomiting | 3 | 0 |
| Alopecia | 0 | 1 |
| Fatigue | 1 | 0 |
| Neuropathy | 1 | 0 |
| Infection | 1 | 0 |

Toxicities > grade 2 shown for patients in both groups.

had not received tamoxifen at the time of initial DTIC treatment, one patient had SD and three PD in response to DBPT. The median number of DBPT courses given was 4 (range 1–6). Median survival was 143 days from commencing DBPT treatment (Table 2).

The two groups were compared to each other to determine whether there were any differences in prognostic factors, either at presentation or at the start of DBPT chemotherapy, and for differences in response or survival. Apart from there being a predominance of females in the direct switch group ($P < 0.03$; Table 1) there were no differences in prognostic factors, either at primary presentation or at the time of commencing DBPT chemotherapy (Table 1). The time since stopping DTIC treatment was significantly longer in the delayed switch group, reflecting the selection criteria for the two groups (Table 1). There were no significant differences between the two groups in survival from starting DBPT (Table 2) or in survival from primary diagnosis (Table 3). Excluding the nine patients who received DTIC as adjuvant and comparing the survival of patients who received DTIC for metastatic disease, survival from commencing DTIC was significantly longer in the delayed switch group ($P < 0.0004$, Table 3 and Figure 1), than in the directly switched group.

In general the chemotherapy was well tolerated. The significant toxicities were neutropenia: > CTC grade 2 in four patients; thrombocytopenia: > CTC grade 2 in two patients and nausea and vomiting: > CTC grade 2 in three patients (Table 4).

### DISCUSSION

The overall response rate of the 35 patients to DBPT was low, with two patients attaining PRs, and five SD. This suggests that the DBPT regimen is not an effective second-line therapy for DTIC resistant disease. In order to examine further whether this was the case, the patients were analysed in two groups: those with disease refractory to DTIC who were directly changed to the DBPT regimen, and those who were not. There were no responses in the directly switched group and only one patient attained short-lived disease stabilization. Thus no patients with de novo DTIC resistance were sensitive to the combination regimen. The implications of the study for this group of patients are clear. The DBPT regimen is unlikely to benefit patients whose disease is refractory to DTIC therapy.

The group whose switch to DBPT was delayed were more heterogeneous than the directly switched group. It included five patients who had attained a response or disease stabilization with DTIC, patients who had not responded to DTIC in the past, but because of the slow pace of disease had not changed directly to combination therapy, and patients who had received adjuvant DTIC. Two patients attained short-lived PRs and four patients attained SD in response to DBPT. Although this suggests that the DBPT regimen may have a place for such patients, overall survival from commencing DBPT was no different from the directly switched group.

The survival from the time of receiving DTIC for metastatic disease was significantly longer in patients in the delayed switch group than in the directly switched group. This group was likely to have had differences in tumour biology compared to the directly switched group, since they responded or developed stable disease with DTIC treatment and/or had more indolent disease. Nonetheless, there were no conventional prognostic features to distinguish the groups. In the delayed switch group there were patients with tumours that were primarily refractory to DTIC, as in the first group, and those that were not. Mechanisms producing primary resistance to DTIC may differ from those associated with later emergence of resistance. Tests to identify patients with non-refractory disease and the mechanisms of drug resistance would be useful. DTIC is presumed to exert cytotoxic effects by guanine methylation, hence possible factors include differences in DNA repair mechanisms, both for $O^6$-methyl guanine and DTIC induced DNA strandbreaks, for which we and others have found evidence for variability in melanoma (Lee et al, 1991; Saunders et al, 1997; Houlbrook et al, 1998).

There are few studies of patients with metastatic melanoma receiving second-line chemotherapy with conventional cytotoxic agents (Everall and Dowd, 1979; Porcile et al, 1979; Quagliana et al, 1984; Johnson et al, 1985; Mulder et al, 1990; Bajetta et al, 1995; Rusciani et al, 1997). These have generally been phase II studies of heterogeneous groups of patients, only some of whom had previously received DTIC. With one exception, we were unable to find any studies containing comparable numbers of patients to those reported here. In that study, patients refractory to cisplatin and tamoxifen were changed to the DBPT regimen. There were no responses in the 12 patients treated in this way (McClay et al, 1993*b*). The same group showed that synergy between tamoxifen and cisplatin was lost if melanoma cells had previously been

© 2000 Cancer Research Campaign exposed and developed resistance to tamoxifen (McClay et al, 1993*a*). All but four patients in the current study had previously received tamoxifen before receiving cisplatin in the DBPT regimen. It is therefore possible that this affected any potential in vivo synergy between tamoxifen and cisplatin. It is conceivable that this contributed to the low response rate to DBPT, although both patients that responded to DBPT had previously received tamoxifen.

In melanoma, the response rate to the DBPT combination regimen is reported to exceed 40% (McClay and McClay, 1996). The response rate to DTIC is around 20% (Mastrangelo et al, 1992). Hence it has been proposed that DBPT or similar combination regimens are preferable to single-agent DTIC (Reintgen and Saba, 1993; McClay and McClay, 1994). Nonetheless, others reported response rates to DBPT comparable to DTIC (Johnston et al, 1998; Margolin et al, 1998). Recent results of randomized comparison of DTIC to DBPT indicate no significant differences in response rate or survival between the two regimens (Saxman et al, 1999). Differing patient populations could explain the differences in response rates to DBPT outlined above. Furthermore, it is not apparent that higher response rates in metastatic melanoma translate to better symptom palliation or survival (Dorval et al, 1999).

The approach used in this study was, once resistance had been demonstrated to single-agent therapy then drugs which are postulated to synergize with that agent were added to the treatment regimen. The study provided similar results to the randomized comparison of DTIC versus DBPT (Saxman et al, 1999). This approach represents a rapid way of assessing for possible drug synergy and/or cross-resistance before or in parallel with randomized trials.

In conclusion, in patients with progressive metastatic melanoma that is refractory to DTIC treatment, there appears to be no advantage in changing to the DBPT regimen. If patients are to be considered for second-line treatment, identifying patterns of in vitro cross-resistance using newly developed cellular chemosensitivity assays (Cree and Kurbacher, 1997) may suggest other therapeutic possibilities. In patients who have had responses to DTIC or the disease is less aggressive, resistance mechanisms may differ, and there may be a place for combination chemotherapy.

### REFERENCES

Bajetta E, Buzzoni R and Vicario G (1995) Combined carboplatin and cytosine arabinoside in metastatic melanoma refractory to dacarbazine. *Tumori* **81**: 238–240

Braybrooke J, Houlbrook S, Crawley JE, Propper DJ, O'Byrne K, Stratford IJ, Harris AL, Shuker DE and Talbot DC (2000) Evaluation of the alkaline comet assay and urine 3-methyl adenine excretion for monitoring DNA damage in melanoma patients treated with dacarbazine and tamoxifen. *Cancer Chemother Pharmacol* **45**: 111–119

Cree IA and Kurbacher CM (1997) Individualizing chemotherapy for solid tumors: is there any alternative? *Anti-Cancer Drugs* **8**: 541–548

Del Prete SA, Maurer LH, O'Donnell J, Forcier RJ and LeMarbre P (1984) Combination chemotherapy with cisplatin, carmustine, dacarbazine, and tamoxifen in metastatic melanoma. *Cancer Treatment Reports* **68**: 1403–1405

Dorval T, Negrier S, Chevreau C, Avril MF, Baume D, Cupissol D, Oskam R, de Peuter R, Vinke J, Herrera A and Escudier B (1999) Randomized trial of treatment with cisplatin and interleukin-2 either alone or in combination with interferon-alpha-2a in patients with metastatic melanoma: a Federation Nationale des Centres de Lutte Contre le Cancer Multicenter, parallel study. *Cancer* **85**: 1060–1066

Everall JD and Dowd PM (1979) Use of combination chemotherapy with CCNU, bleomycin, and vincristine in the treatment of metastatic melanoma in patients resistant to DTIC therapy. *Cancer Treatment Reports* **63**: 151–155

Gehan A (1961) The determination of the number of patients required in a preliminary and a follow-up trial of a new chemotherapeutic agent. *J Chronic Dis* **13**: 346–353

Hill GD, Krementz ET and Hill HZ (1984) Dimethyl triazeno imidazole carboxamide and combination therapy for melanoma. IV. Late results after complete response to chemotherapy (Central Oncology Group protocols 7130, 7131, and 7131A). *Cancer* **53**: 1299–1305

Johnson DH, Presant C, Einhorn L, Bartolucci AA and Greco FA (1985) Cisplatin, vinblastine, and bleomycin in the treatment of metastatic melanoma: a phase II study of the Southeastern Cancer Study Group. *Cancer Treatment Reports* **69**: 821–824

Johnston SR, Constenla DO, Moore J, Atkinson H, A'Hern RP, Dadian G, Riches PG and Gore ME (1998) Randomized phase II trial of BCDT [carmustine (BCNU), cisplatin, dacarbazine (DTIC) and tamoxifen] with or without interferon alpha (IFN-alpha) and interleukin (IL-2) in patients with metastatic melanoma. *Br J Cancer* **77**: 1280–1286

Lee SM, Thatcher N and Margison GP (1991) O6-alkylguanine-DNA alkyltransferase depletion and regeneration in human peripheral lymphocytes following dacarbazine and fotemustine. *Cancer Res* **51**: 619–623

Margolin KA, Liu PY, Flaherty LE, Sosman JA, Walker MJ, Smith JW, 3rd, Fletcher WS, Weiss GR, Unger JM and Sondak VK (1998) Phase II study of carmustine, dacarbazine, cisplatin, and tamoxifen in advanced melanoma: a Southwest Oncology Group study. *J Clin Oncol* **16**: 664–669

Mastrangelo MJ, Bellet RE, Kane MJ and Berd D (1992) Chemotherapy of melanoma. In: *Chemotherapy Source Book,* Perry MC (ed), pp. 886–907. Williams and Wilkins: Baltimore.

McClay EF and McClay ME (1994) Tamoxifen: is it useful in the treatment of patients with metastatic melanoma? *J Clin Oncol* **12**: 617–626

McClay EF and McClay ME (1996) Systemic chemotherapy for the treatment of metastatic melanoma. *Semin Oncol* **23**: 744–753

McClay EF, Albright KD, Jones JA, Christen RD and Howell SB (1993*a*) Tamoxifen modulation of cisplatin sensitivity in human malignant melanoma cells. *Cancer Res* **53**: 1571–1576

McClay EF, McClay ME, Albright KD, Jones JA, Christen RD, Alcaraz J and Howell SB (1993*b*) Tamoxifen modulation of cisplatin resistance in patients with metastatic melanoma. A biologically important observation. *Cancer* **72**: 1914–1918

Mulder NH, Sleijfer DT, de VE, Schraffordt KH and Willemse PH (1990) Phase II study of carboplatin and cytosine arabinoside in patients with disseminated malignant melanoma. *J Cancer Res Clin Oncol* **116**: 301–302

Philip PA, Carmichael J, Tonkin K, Ganesan TA and Harris AL (1994) A phase II study of high-dose hydroxyurea and dacarbazine (DTIC) in the treatment of metastatic malignant melanoma. *Eur J Cancer* **30**: 1027–1029

Porcile G, Musso M, Boccardo F, Rosso R and Santi L (1979) Combination chemotherapy with vinblastine, bleomycin and methotrexate in DTIC-resistant metastatic melanoma. *Tumori* **65**: 237–240

Propper D, Macaulay V, O'Byrne KJ, Braybrooke J, Wilner SM, Ganesan TS, Talbot DC and Harris AL (1998) A phase II study of bryostatin 1 in metastatic malignant melanoma. *Br J Cancer* **78**: 1337–1341

Quagliana JM, Stephens RL, Baker LH and Costanzi JJ (1984) Vindesine in patients with metastatic malignant melanoma: a Southwest Oncology Group study. *J Clin Oncol* **2**: 316–319

Reintgen D and Saba H (1993) Chemotherapy for stage 4 melanoma: a three-year experience with cisplatin, DTIC, BCNU, and tamoxifen. *Semin Surg Oncol* **9**: 251–255

Rusciani L, Petraglia S, Alotto M, Calvieri S and Vezzoni G (1997) Postsurgical adjuvant therapy for melanoma. Evaluation of a 3-year randomized trial with recombinant interferon-alpha after 3 and 5 years of follow-up. *Cancer* **79**: 2354–2360

Saunders MP, Salisbury AJ, O'Byrne KJ, Souliotis VL, Varcoe SM, Talbot DC, Kyrtopoulos SA and Harris AL (1997) A phase II study evaluating the effect of tamoxifen on DNA repair in melanoma patients treated with dacarbazine. *Anticancer Res* **17**: 4677–4680

Saxman SB, Meyers ML, Chapman PP, Destro AN, Panageas KS, Begg CB, Monaco F, Agarwala SS, Schuchter LM, Ernstoff MS, Einhorn LH, Kirkwood JM and Houghton AN (1999) A phase III multicenter randomized trial of DTIC, cisplatin, BCNU and tamoxifen versus DTIC alone in patients with metastatic melanoma. *Proc Annu Meet Am Soc Clin Oncol* **18**: 2068A

© 2000 Cancer Research Campaign 
